# Identification of a Specific Gene Module for Predicting Prognosis in Glioblastoma Patients

**DOI:** 10.3389/fonc.2019.00812

**Published:** 2019-08-27

**Authors:** Xiangjun Tang, Pengfei Xu, Bin Wang, Jie Luo, Rui Fu, Kuanming Huang, Longjun Dai, Junti Lu, Gang Cao, Hao Peng, Li Zhang, Zhaohui Zhang, Qianxue Chen

**Affiliations:** ^1^Department of Neurosurgery, Renmin Hospital of Wuhan University, Wuhan, China; ^2^Department of Neurosurgery, Taihe Hospital, Hubei University of Medicine, Shiyan, China; ^3^Department of Neurosurgery, Affiliated Hospital of Xi'an Jiaotong University Health Science Center, Xi'an, China; ^4^Department of Neurology, Renmin Hospital of Wuhan University, Wuhan, China

**Keywords:** glioblastoma, WGCNA, prognostic model, cox proportional hazards regression model, nomogram

## Abstract

**Introduction:** Glioblastoma (GBM) is the most common and malignant variant of intrinsic glial brain tumors. The poor prognosis of GBM has not significantly improved despite the development of innovative diagnostic methods and new therapies. Therefore, further understanding the molecular mechanism that underlies the aggressive behavior of GBM and the identification of appropriate prognostic markers and therapeutic targets is necessary to allow early diagnosis, to develop appropriate therapies and to improve prognoses.

**Methods:** We used a weighted gene co-expression network analysis (WGCNA) to construct a gene co-expression network with 524 glioblastoma samples from The Cancer Genome Atlas (TCGA). A risk score was then constructed based on four module genes and the patients' overall survival (OS) rate. The prognostic and predictive accuracy of the risk score were verified in the GSE16011 cohort and the REMBRANDT cohort.

**Results:** We identified a gene module (the green module) related to prognosis. Then, multivariate Cox analysis was performed on 4 hub genes to construct a Cox proportional hazards regression model from 524 glioblastoma patients. A risk score for predicting survival time was calculated with the following formula based on the top four genes in the green module: risk score = (0.00889 × EXP_CLEC5A_) + (0.0681 × EXP_FMOD_) + (0.1724 × EXP_FKBP9_) + (0.1557 × EXP_LGALS8_). The 5-year survival rate of the high-risk group (survival rate: 2.7%, 95% CI: 1.2–6.3%) was significantly lower than that of the low-risk group (survival rate: 8.8%, 95% CI: 5.5–14.1%).

**Conclusions:** This study demonstrated the potential application of a WGCNA-based gene prognostic model for predicting the survival outcome of glioblastoma patients.

## Introduction

Glioma is one of the most common types of malignant brain tumors and has a very poor prognosis (1). The efficacy of conventional surgery plus radio- and chemotherapy is poor. Several signature molecular markers have been used in the diagnosis, therapy and prognosis of glioma. For example, methyl guanine methyl transferase (MGMT) promoter methylation is considered a predictive marker for the resistance of glioblastoma (GBM) to chemotherapy with temozolomide (2). The 1p/19q co-deletion is a molecular signature of oligodendroglial tumors and a predictive marker for the response of anaplastic gliomas to vincristine (PCV) chemotherapy. High WT-1 expression is significantly associated with worse outcomes in diffuse astrocytic tumors. IDH1/IDH2 mutations have a strong favorable prognostic value across all glioma histopathological grades (3–5). With the advancement of gene technology, molecular signatures for the classification of gliomas have become prominent in recent years. The 2016 revision of the World Health Organization (WHO) classification of tumors of the central nervous system (6) includes novel classes of diffuse gliomas based on genomic features. Though molecular diagnostics increase diagnostic accuracy and prognostic yield compared to previous histology-based classifications, the current clinical prediction and treatment outcomes are still not satisfactory (7). As GBM is notoriously heterogeneous and complex, multi-parameter markers are much more accurate for cancer prognosis than a single biomarker. Therefore, a proper analytical model is highly desirable.

In the present study, we identified gene modules related to the overall survival (OS) and recurrence time of GBM based on The Cancer Genome Atlas (TCGA) database and weighted gene co-expression network analysis (WGCNA). The TCGA database contains genomic expression, sequence, methylation, and copy number variation data on over 11,000 individuals and over 30 kinds of cancers (8, 9). WGCNA is based on a system of biological methods for describing the correlation patterns among genes and modules of highly correlated genes. By using Kaplan-Meier survival analysis and multivariate Cox regression analysis, we identified a prognostic model for GBM patients based on gene characteristics. Our findings may provide novel insight toward developing a promising predictive tool for the prognosis of GBM.

## Materials and Methods

### Patients

**Figure 1:**
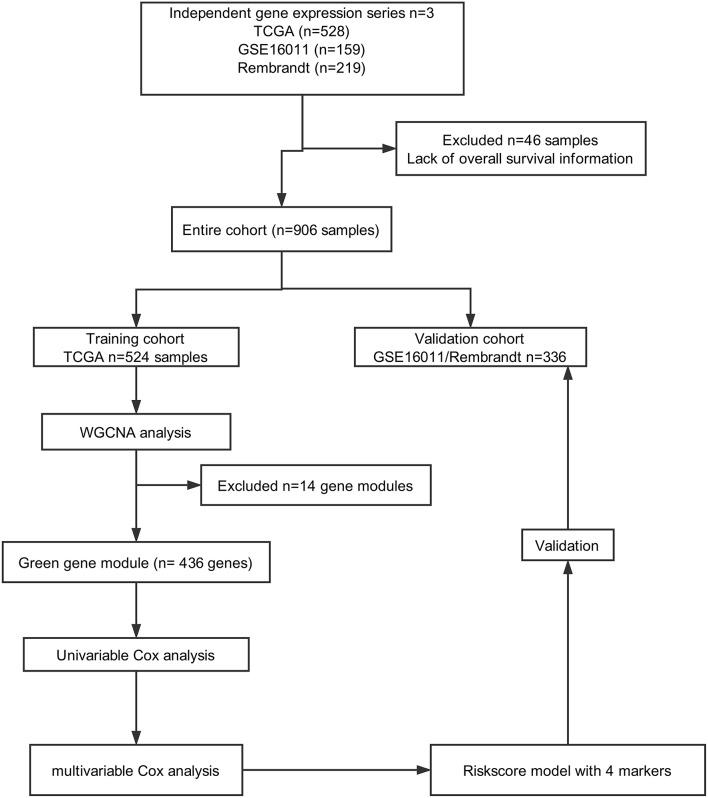
Flow chart of data collection and analysis.

A total of 906 glioma cases were collected from three databases in this study, including 528 samples from TCGA (https://portal.gdc.cancer.gov), 219 samples from REMBRANDT (https://gdoc.georgetown.edu/gdoc/), and 159 samples from the GSE16011 dataset (http://www.ncbi.nlm.nih.gov/geo/query/acc.cgi?acc=GSE16011). Forty-six samples were excluded due to a lack of OS information. As shown in Figure 1, we grouped cases from TCGA into a training cohort, whereas all cases from REMBRANDT and GSE16011 were used for validation.

### Data Pre-processing

Microarray data of the 906 samples were normalized by the affy package. All data were filtered to reduce outliers. For genes with several probes, the median of all probes was chosen. For probes with missing values, the impute package (http://bioconductor.org/packages/release/bioc/html/impute) was used to fill the missing values. Finally, 12,700 genes were obtained from the TCGA dataset.

### Construction of the Weighted Gene Co-expression Network

By choosing 6 as a soft threshold, a weighted gene co-expression network was constructed using the R package WGCNA (10), which has the approximate scale-free fundamental property of the biological gene networks. A co-expression similarity matrix was composed of the absolute value of the correlation between the expression levels of transcripts. The network modules were generated using the topological overlap measure (TOM) (11), and the dynamic hybrid cut method (a bottom-up algorithm) was used to identify co-expression gene modules (12). Finally, the modules with highly correlated genes were merged, and the minimum height for merging modules was set to 0.2. Gene significance (GS) and module significance (MS) were calculated to measure the correlation between the sample traits (recurrence time, CpG island methylator phenotype (CIMP) status, survival time, status, IDH1 status, MGMT status, subtype, age and sex) of either the genes or modules. The targeted module genes were visualized with Cytoscape 3.5.1 software (13).

### Functional Enrichment Analysis

The biological process (BP) ontology of the modules was analyzed by Gene Ontology (GO) (14), while pathway enrichment was analyzed by the Kyoto Encyclopedia of Genes and Genomes (KEGG) (15). The function of module genes was verified by the R package clusterProfiler (16). The corrected *P*-value (false discovery rate, FDR) < 0.05 was identified as a significant outcome.

### Identification of the Predicted Survival of Glioblastoma Patients by the Cox Proportional Hazards Regression Model

To verify the significance of the genes screened above, the 436 green module genes were first screened using univariate Cox proportional hazards regression, and the 230 genes with *p*-value <0.05 was selected for the advanced analysis (Supplemental Data 2). According to the *p*-value, we selected only the top 14 survival-related genes for visualization using the R package forestplot. Then, a multivariate Cox regression model analysis was performed to establish a Cox proportional hazards regression prognostic model, which was calculated as follows: risk score = Σ(C × EXPgene), where EXP was the mRNA expression of the crucial gene, and C was the regression coefficient for the corresponding gene in the multivariate Cox hazard model analysis. The optimal model was determined based on akaike information criterion (AIC). The relevant codes were provided in the Supplemental File. The samples were divided into a high-risk group and a low-risk group according to the median risk score of the training dataset from TCGA.

### Statistical Analysis

Survival curves were constructed by the Kaplan-Meier method and compared by the log-rank test, which was carried out through the R package survival. The sensitivity and specificity of the survival prediction based on the risk score were depicted by a time-dependent receiver operating characteristic (ROC) curve using the R package survivalROC. Gene set enrichment analysis (GSEA) was used to identify the pathways that were significantly enriched between the high- and low-risk groups. The Cox regression model was used to perform the multivariable survival analysis and generate nomograms. Calibration curves were used to assess whether the actual outcomes approximately predicted outcomes for the nomogram. Nomogram and calibration curves were performed with the rms package (https://CRAN.R-project.org/package=rms). The discrimination of the nomogram was measured and compared by the C-index. All statistical tests were two-sided, and *P* < 0.05 was considered statistically significant. Statistical analyses were conducted using R software (version 3.4.3, www.r-project.org).

## Results

### Pre-processing of RNA Sequence Data and Clinical Data

In total, 906 glioblastoma microarray and clinical data were downloaded from TCGA, REMBRANDT and GSE16011. We constructed an mRNA expression matrix with gene symbols and patient barcodes. Furthermore, outlier samples with expression quantities <20% were screened. A total of 46 samples were discarded owing to the lack of OS information. Finally, the top 5,000 genes with the greatest variance obtained from the training cohort were used in the WGCNA studies.

### Identification of Modules Associated With Glioma Survival Status

**Figure 2:**
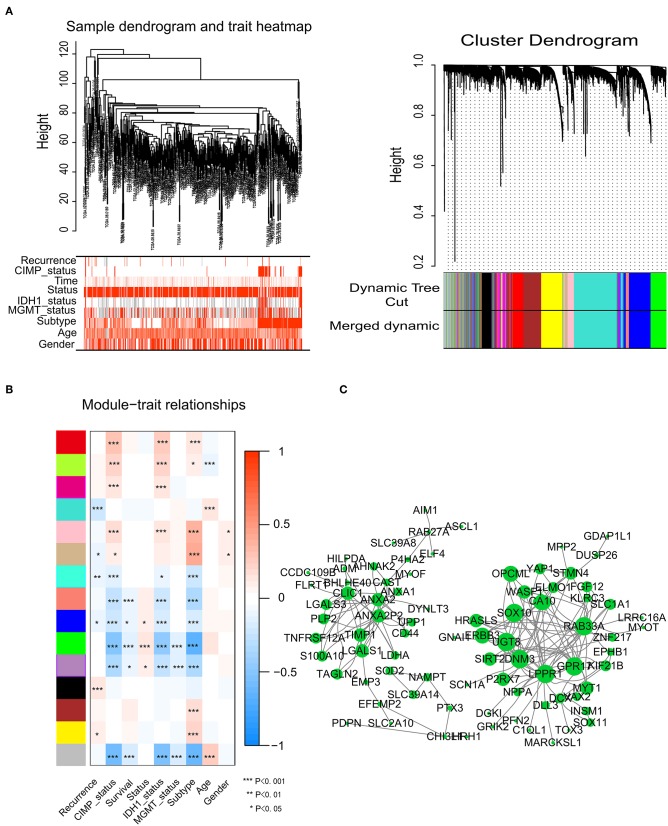
Network construction of the weighted co-expressed genes and their associations with clinical traits. **(A)** Hierarchical clustering tree of the TCGA-GBM samples based on the training cohort. Dendrogram tips are labeled with the TCGA-GBM unique name. In the hierarchical dendrogram, lower branches correspond to higher co-expression. The branches of the cluster dendrogram correspond to the 15 different gene modules based on topological overlaps. Each piece of the leaves on the cluster dendrogram represents a gene. **(B)** Module-trait relationships. The background colors of the numbers represent the strength of the correlation between the gene module and the clinical traits, which increased from blue to red. Each column corresponds to a clinical trait. **(C)** Visualization of the co-expression network of the green module. The larger the nodes and the numerous edges, the more significant the gene is. Based on weight, not all genes were represented.

To identify significant gene modules, we constructed a gene co-expression network with WGCNA. With a scale-free network and topological overlaps, we generated a hierarchical clustering tree based on the dynamic hybrid cut (Figure 2A). Finally, 15 gene modules were identified, and the branches of the tree represent different gene modules. The non-co-expressed genes were included in the “gray” module, which was not further analyzed (Figure 2B). The relationships of the fifteen modules were analyzed with clinical traits, such as survival time, recurrence time, age, and sex. The green module correlated significantly with survival status (Figure 2B). A total of 436 genes were included in the green module.

### Visualization of Green Module Genes

Network screening was used to detect the hub genes in the green module. The co-expression network of the green module was visualized with a Cytoscape graph. As shown in Figure 2C, the hub genes were centrally located in the modules and may be the key elements of the modules. The larger the nodes and the numbers of the edges, the more significant the gene is. When depicted based on weight, not all genes were represented.

### Functional Enrichment Analysis

**Figure 3:**
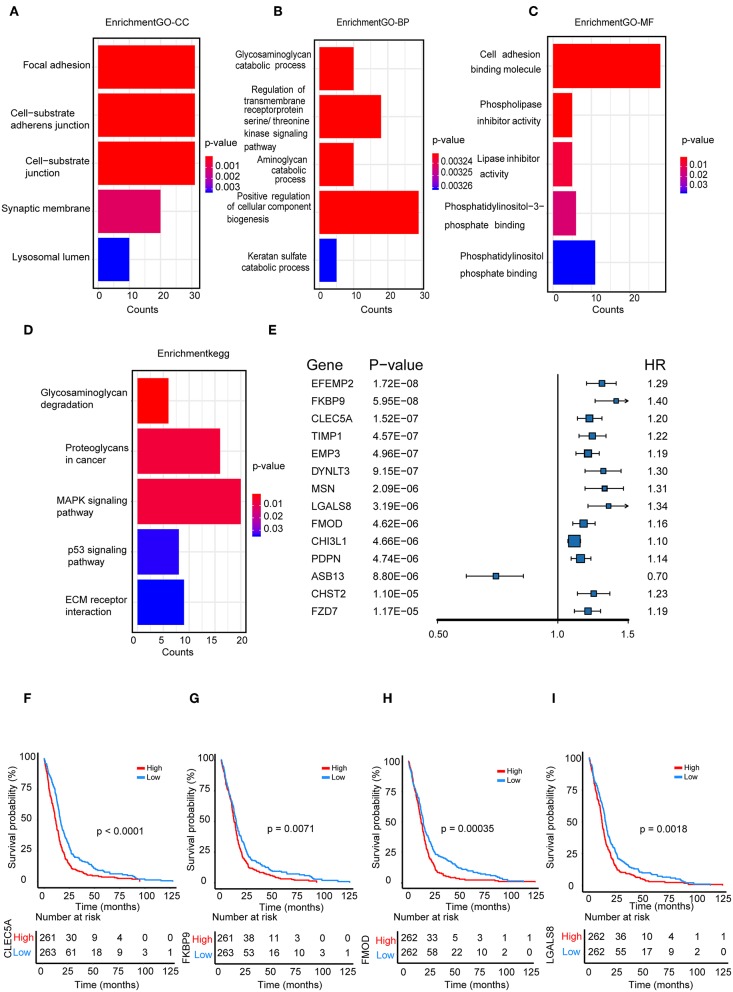
Functional enrichment analysis. **(A)** Biological process **(B)** cellular component, **(C)** molecular function; **(D)** enrichment of Kyoto Encyclopedia of Genes and Genomes (KEGG) pathway analysis for hub genes related to survival time; **(E)** The top 14 genes which were significantly related to survival time in univariate analysis; **(F–I)** Kaplan-Meier curves for CLEC5A,FKBP9, FMOD, and LGALS8 in the TCGA cohort.

We performed a functional enrichment analysis of the green module using GO analysis. As shown in Figures 3A–D, enriched BPs were mainly involved in the positive regulation of cellular component biogenesis. The cellular components (CCs) were mainly enriched in focal adhesion and the cell substrate adherens junction. Enriched molecular functions (MFs) were mainly involved in cell adhesion molecule binding. KEGG pathway analysis showed that the MAPK signaling pathway was the most enriched pathway, followed by proteoglycans in cancer and the regulation of the actin cytoskeleton. The results suggested that these genes were closely related to cell adhesion function.

### Identification and Validation of a Cox Proportional Hazards Regression Model

We further selected all genes of the green module to perform a univariate Cox analysis (Figure 3E). Then, multivariate Cox analysis was performed on the four genes that were significantly related to survival time. A Cox proportional hazards regression model was constructed with the TCGA cohort. The risk score for predicting survival time was calculated with the following formula based on the four genes: risk score = (0.00889 × EXP_CLEC5A_) + (0.0681 × EXP_FMOD_) + (0.1724 × EXP_FKBP9_) + (0.1557 × EXP_LGALS8_).

**Figure 4:**
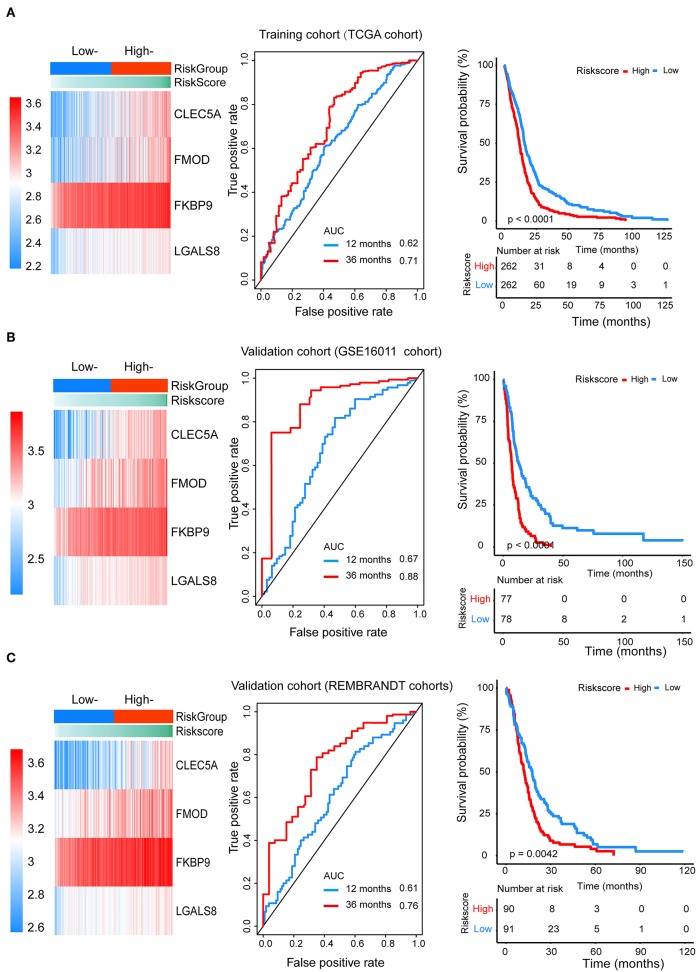
The prognostic efficiency of the Cox proportional hazards regression model. Heat map of the model genes in **(A)** training set of the TCGA, **(B)** test set of GSE16001, **(C)** test set of Rembrandt; ROC curves of the four genes signature for predicting 12- and 36-months survival of glioblastoma. The 12- and 36-months areas (AUC) under the ROC curves indicate higher predictive value; Kaplan–Meier curves analyze the survival of the high-risk group and the low-risk group, the high-risk group had the worse outcome (*P* < 0.001).

We divided patients from the training set into high-risk (n = 262) and low-risk (n = 262) groups according to the median of the risk score. The 1- and 3-year areas under the ROC curve were 0.62 and 0.71, respectively, indicating a high predictive value. Additionally, the predictive model can function as a good predictive indicator of the survival of glioma patients, which was confirmed by Kaplan-Meier curves. Patients with high-risk scores exhibited worse OS according to the Kaplan-Meier curves. The 5-year and 3-year survival rates of the high-risk group (2.7 and 6.8%, respectively) were significantly worse than those of the low-risk group (8.8 and 18.9%, respectively; Figure 4A). Moreover, the Kaplan-Meier curves confirmed that the four genes could function as predictive indicators for the survival of GBM patients in the training cohort (Figures 3F–I).

Furthermore, we assessed the prognostic effect of different clinical characteristics using a univariate Cox proportional hazards regression model. The results showed that CIMP status, IDH1 status, MGMT status, age, and risk score were associated with OS (*P* < 0.01) (Table 1). However, the multivariate regression model showed that the risk score and age were independent prognostic factors associated with OS.

**Table 1:** The prognostic effect of different clinical characteristics.

	**Univariate analysis**^**a**^			**Multivariate analysis**^**b**^		
	**HR**	**95%CI**	***P*-value**	**HR**	**95%CI**	***P*-value**
CIMP-status	0.35	0.24–0.5	<0.001	0.29	0.04–2.19	0.232
IDH1-status	0.34	0.21–0.55	<0.001	1.8	0.23–14.08	0.573
MGMT-status	0.69	0.54–0.87	<0.001	0.84	0.64–1.1	0.205
Subtype	0.93	0.86–1.01	0.07	-	-	-
Age	1.03	1.03–1.04	<0.001	1.03	1.02–1.04	<0.001
Gender	1.16	0.96–1.41	0.13	-	-	-
Risk score	1.57	1.3–1.89	<0.001	1.49	1.14–1.94	0.003

a These data were used to perform the Cox proportional hazards regression.

b *Multivariate analysis used stepwise addition of clinical covariates related to survival in univariate analysis (P < 0.01) and the ultimate models contained those covariates that were significantly associated with survival (P < 0.01)*.

**Figure 5:**
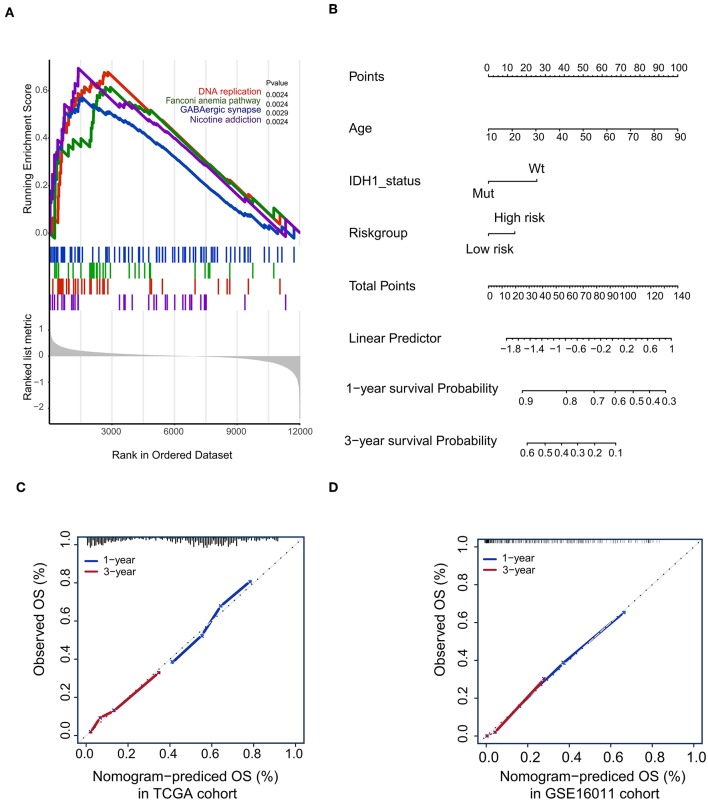
Gene-set enrichment analysis (GSEA) and Nomogram. **(A)** The GSEA showed that high-risk group highly enriched in Base excision repair, Cell cycle, DNA replication, Ribosome; **(B)** Nomogram to predict the 1- and 3-year OS. Calibration curve for OS nomogram model in the TCGA cohort **(C)** and GSE16011 cohort **(D)**.

To confirm that the proposed risk score model has similar prognostic value in different populations, the same formula was applied to the GSE16011 and REMBRANDT cohorts. The results showed that patients in the high-risk group had a significantly lower OS rate than those in the low-risk group in both the GSE16011 and REMBRANDT cohorts (Figures 4B–C). The functional GSEA showed that the high-risk group was highly enriched in genes closely related to base excision repair, the cell cycle, DNA replication, and ribosome function (Figure 5A).

### Construction of a Predictive Nomogram

To develop a quantitative method to predict patients' OS rate, we constructed a nomogram in the TCGA cohort. The risk score was stratified into high- and low-risk groups based on the median. The predictors included age, risk group, and IDH1 status (Figure 5B). Due to the lack of IDH1 mutation information in the REMBRANDT cohorts, the calibration curves for the 1- and 3-year OS rates were well-predicted in only the TCGA and GSE16011 cohorts (C-index: 0.65 for the TCGA cohort and 0.68 for the GSE16011 cohort; Figures 5C,D).

## Discussion

Gliomas are the most common and malignant brain tumors with poor prognosis, especially GBM. The most promising treatments, such as surgery, radiation, and chemotherapy with temozolomide, improve survival measured in only weeks rather than years (17). Precise studies of GBM biology and molecular markers have renewed our understanding of GBM. In 2008, Parsons et al. first proposed subtypes of GBM based on specific gene alterations (18). In 2016, the WHO revised the classification of tumors of the central nervous system based on gene technology and molecular signatures. The classification contained some well-known biomarkers, such as MGMT methylation, 1p/19q co-deletion, IDH 1 or 2, and EGFR. Recently, Suchorska et al. reported that amino acid positron emission tomography (PET)-based metabolic imaging can be used as a promising tool for the non-invasive characterization of molecular features and to provide additional prognostic information (19). These classifications and studies helped with prognosis, survival time, and response to treatment. As GBMs are heterogeneous and complex, molecular signatures are superior to single biomarkers in the prognosis of glioma.

To identify a gene signature associated with the survival status of GBM patients, we first constructed a weighted gene co-expression network in 524 glioma samples and generated the survival time-specific green module. The detected hub genes in the green module were significantly correlated with the survival status of patients with GBM. The GO and KEGG functional enrichment analysis showed that the genes that were closely related to adhesion function, adhesion molecules and the MAPK signaling pathway accounted for the highest proportion of green module genes. Adhesion function is a key factor in glioma invasiveness, and adhesion molecules play an important role in gliomagenesis. The MAPK pathway regulates the activity of transcription factors that function in proliferation, survival, differentiation, and apoptosis (20). Furthermore, this signaling pathway is also activated by EGFR signaling. The MAPK pathway could also be directly or indirectly activated through mutations of downstream components. In high-grade gliomas, MAPK-activated samples presented prolonged survival in comparison to other high-grade tumors. In low-grade gliomas, the presence of activated MAPK was also a predictor of favorable patient outcome, regardless of fusion or hotspot mutation events (21).

To analyze the relationship between survival time and the hub genes of the green module, we selected 436 genes for univariate Cox analysis. Our survival analysis by constructing a Cox proportional hazards regression model showed that CLEC5A, FMOD, FKBP9, and LGALS8 were highly associated with OS. CLEC5A/MDL-1 is a member of the myeloid C-type lectin family expressed in macrophages and neutrophils, which is strongly associated with the activation and differentiation of myeloid cells and has been implicated in the progression of multiple acute and chronic inflammatory diseases. Research by Batliner et al. suggested that CLEC5A/MDL-1 could activate a signaling cascade that results in the activation of downstream kinases in inflammatory responses (22) and maintain lesional macrophage survival, causing their accumulation (23). Another report showed that Japanese encephalitis virus (JEV) directly interacted with CLEC5A. Additionally, anti-CLEC5A mAb could repair the blood-brain barrier, attenuate neuroinflammation, and protect mice from JEV-induced lethality (24). Recently, R. Chai reported that CLEC5A was also a prognostic biomarker of GBM (25). FKBP9 is a peptidyl–prolyl isomerase and is a member of this protein family. It has been implicated in neurodegeneration, mainly through accelerating fibrillization (26, 27). Fibromodulin (FMOD), as a GBM-upregulated gene, promotes glioma cell migration through its ability to generate the formation of filamentous actin stress fibers. FMOD-induced glioma cell migration is dependent on the integrin-FAK-Src-Rho-ROCK signaling pathway (28). FMOD was also reported to be a prognostic biomarker in GBM (29). LGALS8 plays functional roles in promoting GBM cell proliferation and clonal sphere formation (30). Though CLEC5A and FKBP9 have not been reported in glioma-related studies, their features play important roles in cell metabolism and pathological processes. Further studies are needed to explore their relationship with glioma. Therefore, CLEC5A, FMOD, FKBP9, and LGALS8 could be considered crucial prognostic factors in the OS of glioma patients.

In this study, we constructed a prognostic score model of a four-gene signature. The univariate Cox proportional hazards regression result demonstrated that this four-gene signature, together with CIMP status, IDH1 status, MGMT status, and age, was highly associated with OS. The independent prognostic significance was also verified according to a multivariate regression model. The ability of the four-gene model to predict survival outcomes was further confirmed by the validation cohorts from the REMBRANDT and GSE16011 datasets. To further strengthen the accuracy of the model, we combined age, IDH1 status, and risk group to fit a Cox proportional regression model in the TCGA cohort and used a nomogram for visualization. The calibration curves showed high predictive ability in the TCGA and GSE16011 cohorts. Our analysis showed that the four-gene model is likely a promising and viable prognostic signature for the survival status of glioma patients.

In summary, through the construction of a gene co-expression network with data from the TCGA database, a green module with a survival signature was identified using the WGCNA approach. The hub genes were selected from the green module genes and visualized with Cytoscape. By constructing a Cox proportional hazards regression model, four genes were finally identified and used in univariate and multivariate Cox analyses, thereby composing a four-gene module with the risk score = (0.00889 × EXP_CLEC5A_) + (0.0681 × EXP_FMOD_) + (0.1724 × EXP_FKBP9_) + (0.1557 × EXP_LGALS8_). This four-gene module represents a promising and viable prognostic signature for the survival outcome of GBM patients. The present study revealed the potential application of a WGCNA-based gene prognostic model for predicting the survival outcomes of GBM patients.

## Data Availability

Publicly available datasets were analyzed in this study. This data can be found here: https://portal.gdc.cancer.gov.

## Ethics Statement

Ethical approval was waived since we used only publicly available data and materials in this study.

## Author Contributions

XT, PX, and LZ: conception and design. XT, PX, BW, and JLuo: acquisition of data. XT, PX, RF, KH, and ZZ: analysis and interpretation of data. XT, LD, and ZZ: writing and review of the manuscript. JLu, GC, HP, LZ, and QC: study supervision. All authors have read and approved the final version of this manuscript.

### Conflict of Interest Statement

The authors declare that the research was conducted in the absence of any commercial or financial relationships that could be construed as a potential conflict of interest.

## Abbreviations

WGCNA: Weighted Gene Co-expression Network Analysis
TCGA: The Cancer Genome Atlas
GEO: Gene Expression Omnibus
GS: gene significance
MS: module significance
EGFR: epidermal growth factor receptor
HR: hazard ratio
EXP: expression
MAPK: mitogen-activated protein kinase
CLEC5A: C-type lectin member 5A
FMOD: fibromodulin
FKBP: FK506-binding proteins
LGALS8: lectin, galactose binding, soluble 8
CIMP: CpG island methylator phenotype
IDH1: Isocitratedehydrogenase 1
IDH2: isocitrate dehydrogenase 2
MGMT: O6-methylguanine-DNA methyltransferase
FPKM: fragments per kilobase per million
TOM: topological overlap measure
GO: gene ontology
CC: cellular component
MF: molecular function
BP: biological process
KEGG: Kyoto Encyclopedia of Genes and Genomes
ROC: receiver operating characteristic curve
AUC: area under the curve
JEV: Japanese Encephalitis Virus
AIC: akaike information criterion.
