# Supplementary figures and images for: Identification of a Specific Gene Module for Predicting Prognosis in Glioblastoma Patients
Source: Front Oncol. 2019 Aug 27;9:812. doi: 10.3389/fonc.2019.00812 (PMC6718733; doi:10.3389/fonc.2019.00812)

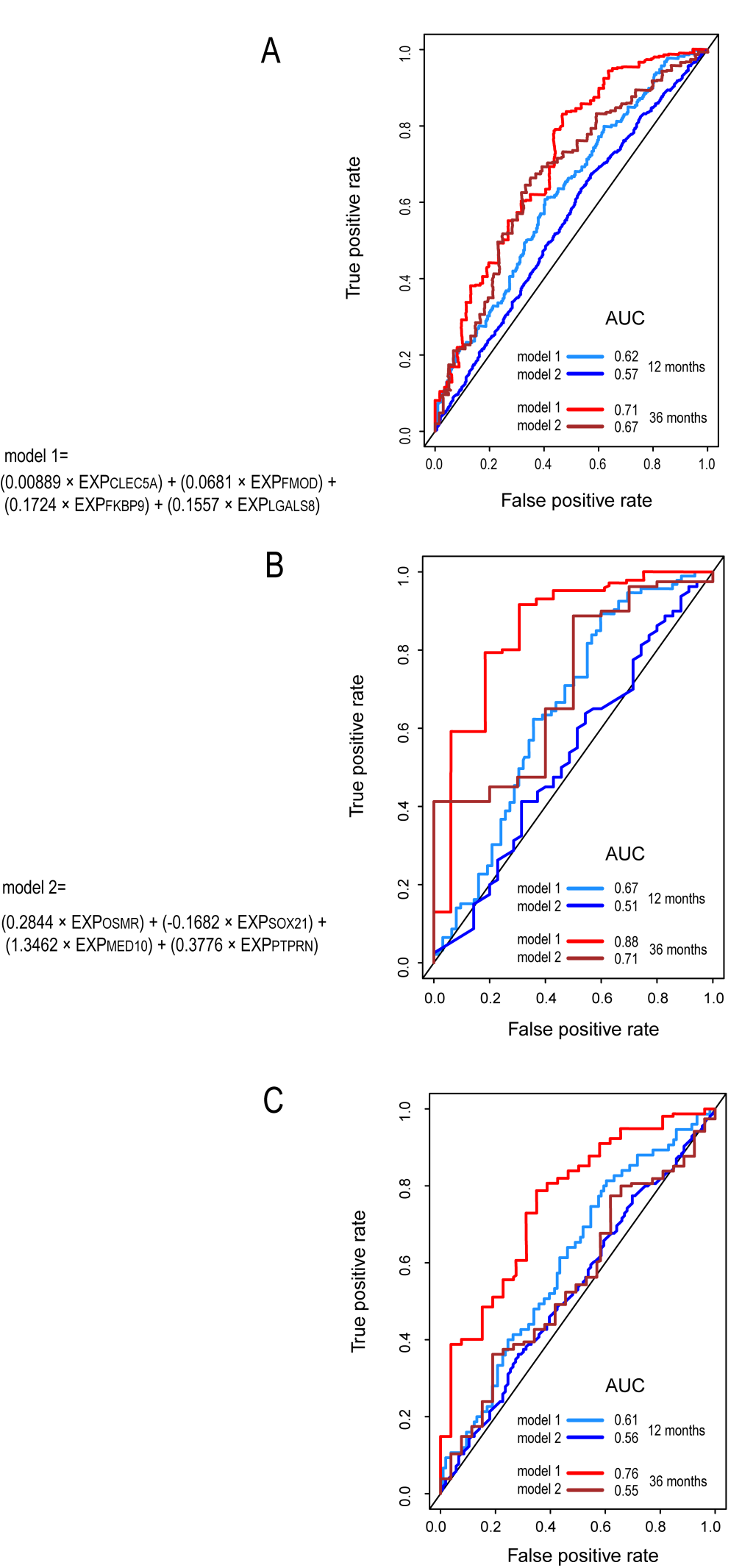


Comparison between different prognostic models

Supplement: Supplementary file 4 [file Data_Sheet_4.docx]
