# Supplementary material for: Identification of a Specific Gene Module for Predicting Prognosis in Glioblastoma Patients
Source: Front Oncol. 2019 Aug 27;9:812. doi: 10.3389/fonc.2019.00812 (PMC6718733; doi:10.3389/fonc.2019.00812)
Supplement: Supplementary file 5 [file Data_Sheet_5.docx]

Codes used for prognostic gene selection and model construction

library("WGCNA")

library("flashClust")

options(stringsAsFactors = FALSE)

expro=read.csv("xxx.csv")

rownames(expro)=expro[,1]

expro=expro[,-1]

expro=t(expro)

expro= expro[apply(expro,1,function(x) sum(x==0))<ncol(expro)*0.8,]

datExpr = t(expro[order(apply(expro,1,mad), decreasing = T)[1:5000],])

dim(datExpr)

gsg = goodSamplesGenes(datExpr,verbose =3);

gsg$allOK

sampleTree = hclust(dist(datExpr), method = "average")

plot(sampleTree, main = "Sample clustering to detect outliers"

, sub="", xlab="")

abline(h =130, col = "red")

clust = cutreeStatic(sampleTree, cutHeight =130, minSize = 10)

table(clust)

keepSamples = (clust==1)

datExpr = datExpr[keepSamples, ]

nGenes = ncol(datExpr)

nSamples = nrow(datExpr)

#save(datExpr, file = "dataInput.RData")

rownames(datExpr)

#write.table(datExpr,"datExpr.csv",row.names = T,sep=",")

Read the trait data

samples=read.csv("xxxx.csv");

clustsample=rownames(datExpr)

traitRows = match(clustsample, samples$Sample)

datTraits=samples[traitRows,-1]

rownames(datTraits)=samples[traitRows,1]

#names(datTraits)

sampleTree2=hclust(dist(datExpr),method="average")

traitColors=numbers2colors(datTraits,signed=FALSE,colors=blueWhiteRed(100)[51:100])

plotDendroAndColors(sampleTree2,traitColors,groupLabels=names(datTraits),main=

"Sample dendrogram and trait heatmap",cex.dendroLabels=0.63,autoColorHeight=

FALSE,colorHeight=0.3)

#save(datExpr,datTraits,file = "dataInput.RData")

##pickSoftThreshold##

powers = c(c(1:10), seq(from = 12, to=20, by=2))

sft = pickSoftThreshold(datExpr, powerVector = powers, verbose = 5)

sizeGrWindow(9, 5)

par(mfrow = c(1,2));

cex1 = 0.9;

plot(sft$fitIndices[,1], -sign(sft$fitIndices[,3])*sft$fitIndices[,2],

xlab="Soft Threshold (power)",ylab="Scale Free Topology Model Fit,signed R^2",type="n",

main = paste("Scale independence"));

text(sft$fitIndices[,1], -sign(sft$fitIndices[,3])*sft$fitIndices[,2],

labels=powers,cex=cex1,col="red");

abline(h=0.90,col="red")

plot(sft$fitIndices[,1], sft$fitIndices[,5],

xlab="Soft Threshold (power)",ylab="Mean Connectivity", type="n",

main = paste("Mean connectivity"))

text(sft$fitIndices[,1], sft$fitIndices[,5], labels=powers, cex=cex1,col="red")

ADJ1=abs(cor(datExpr,use="p"))^6

k=as.vector(apply(ADJ1,2,sum,na.rm=T))

sizeGrWindow(10,5)

par(mfrow=c(1,2))

hist(k)

scaleFreePlot(k,main = "Check scale free topology\n")

softPower=6

adjacency=adjacency(datExpr,power=softPower)

TOM=TOMsimilarity(adjacency)

dissTOM=1-TOM

geneTree=flashClust(as.dist(dissTOM),method="average")

sizeGrWindow(12,9)

plot(geneTree,xlab = "",sub="",main = "Gene clustering on TOM-based dissimilarity",labels = FALSE,hang=0.04)

minModuleSize=30

dynamicMods=cutreeDynamic(dendro = geneTree,distM=dissTOM,deepSplit = 2,

pamRespectsDendro = FALSE,minClusterSize = minModuleSize)

dynamicColors=labels2colors(dynamicMods)

table(dynamicColors)

sizeGrWindow(8,6)

plotDendroAndColors(geneTree,dynamicColors,"Dynamic Tree Cut",dendroLabels =

FALSE,hang=0.03,addGuide = TRUE,guideHang =0.05,main= "Gene dendrogram

and module colors")

MEList=moduleEigengenes(datExpr,colors=dynamicColors)

MEs=MEList$eigengenes

MEDiss=1-cor(MEs)

METree=flashClust(as.dist(MEDiss),method="average")

sizeGrWindow(7,6)

plot(METree,main = "Clustering of module eigengenes",xlab = "",sub="")

MEDissThres=0.25

abline(h=MEDissThres,col="red")

merge = mergeCloseModules(datExpr, dynamicColors, cutHeight = MEDissThres, verbose = 3)

mergedColors=merge$colors

mergedMEs=merge$newMEs

sizeGrWindow(12,9)

#pdf(file = "Plots/geneDendro-3.pdf", wi = 9, he = 6)

plotDendroAndColors(geneTree, cbind(dynamicColors, mergedColors),

c("Dynamic Tree Cut", "Merged dynamic"),

dendroLabels = FALSE, hang = 0.03,

addGuide = TRUE, guideHang = 0.05)

moduleColors=mergedColors

colorOrder = c("grey", standardColors(50));

moduleLabels=match(moduleColors,colorOrder)-1

MEs=mergedMEs

save(MEs,moduleLabels,moduleColors,geneTree,file="networkConstruction-stepByStep.RData")

datME=moduleEigengenes(datExpr,moduleColors,trapErrors = FALSE)$eigengenes

signif(cor(datME,use="p"),2)

dissimME=(1-t(cor(datME,method="p")))/2

hclustdatME=hclust(as.dist(dissimME),method="average")

par(mfrow=c(1,1))

plot(hclustdatME,main="Clustering tree based of the module eigengenes")

sizeGrWindow(8,7)

which.module="green"

ME=datME[,paste("ME",which.module,sep = "")]

par(mfrow=c(2,1),mar=c(0.3,5.5,3,2))

plotMat(t(scale(datExpr[,moduleColors==which.module])),nrgcols=30,rlabels=F,rcols=

which.module,main=which.module,cex.main=2)

par(mar=c(5,4.2,0,0.7))

barplot(ME,col=which.module,main="",cex.main=2,ylab = "eigengene expression",xlab = "array sample")

sizeGrWindow(16,18)

datKME = signedKME(datExpr,MEs)

write.table(datKME,"KME.csv", row.names =T, sep = ",")

nGenes = ncol(datExpr)

nSamples = nrow(datExpr)

MEs0 = moduleEigengenes(datExpr, moduleColors)$eigengenes

MEs = orderMEs(MEs0)

moduleTraitCor=cor(MEs,datTraits,use ="p");

moduleTraitPvalue = corPvalueStudent(moduleTraitCor,nSamples);

sizeGrWindow(10,6)

# Will display correlations and their p-values

textMatrix =paste(signif(moduleTraitCor,2),"\n(",signif(moduleTraitPvalue, 1),")", sep = "");

dim(textMatrix) = dim(moduleTraitCor)

par(mar = c(6,8.5,3,3))

# Display the correlation values within a heatmap plot

labeledHeatmap(Matrix = moduleTraitCor,

xLabels = names(datTraits),

yLabels = names(MEs),

ySymbols = names(MEs),

colorLabels = FALSE,

colors = blueWhiteRed(50),

textMatrix = textMatrix,

setStdMargins = FALSE,

cex.text = 0.5,

zlim = c(-1,1),

main = paste("Module-trait relationships"))

library(survminer)

library(survival)

library(survivalROC)

surdata<- read.csv("xx.csv")

HR=data.frame()

for(i in colnames(surdata[,4:ncol(surdata)])){

cox <- coxph(Surv(time, status) ~ surdata[,i], data = surdata)

coxSummary = summary(cox)

HR=rbind(HR,cbind(gene=i,HR=coxSummary$coefficients[,"exp(coef)"],

lower=paste0(round(coxSummary$conf.int[,3],2)),

upper=paste0(round(coxSummary$conf.int[,4],2)),

pvalue=coxSummary$coefficients[,"Pr(>|z|)"]))

}

write.csv(HR,"univercox.csv")

rt=read.csv("xxx.csv")

rownames(rt)=rt[,1]

rt=rt[,-1]

cox <- coxph(Surv(time, status) ~ ., data = rt)

cox=step(cox,direction = "both")
